# The New York Post-Graduate Medical School and Hospital

**Published:** 1888-09

**Authors:** 


The New York Post-Graduate Medical School and Hospital.—During the winter session of 1887–1888, over 335 different physicians attended the courses of instruction at the New York Post-Graduate Medical School and Hospital, 226 East Twentieth street, New York City, an increase of more than sixty per cent. over last year. In the hospital department about 400 operations were performed, all of major importance. To all of these, matriculates had access, as the hospital is used solely as a means of clinical instruction. This remarkable increase in the matriculation list has necessitated both larger clinical space and hospital accommodations, so that a new clinical amphitheatre has been erected and will be used for the first time at the opening of the winter session, September 17, 1888. A new and commodious laboratory has been erected and furnished with the latest apparatus for the study of normal and pathological tissues. The nose and throat clinical room has also been enlarged. Professors Abraham Jacobi, Robert F. Weir, Joseph E. Winters, L. Bolton Bangs, and Peter A. Callan have been appointed to the faculty. The session of 1888–1889 promises to be the most prosperous ever held.

